# Origins and diversity of a cosmopolitan fern genus on an island archipelago

**DOI:** 10.1093/aobpla/plv118

**Published:** 2015-10-20

**Authors:** Paul G. Wolf, Carol A. Rowe, Joshua P. Der, Martin P. Schilling, Clayton J. Visger, John A. Thomson

**Affiliations:** 1 Department of Biology, Utah State University, Logan, UT 84322, USA; 2 Ecology Center, Utah State University, Logan, UT 84322, USA; 3 Department of Biological Science, California State University, Fullerton, CA 92834, USA; 4 Department of Biology, University of Florida, Gainesville, FL 32611, USA; 5 National Herbarium of NSW, Royal Botanic Gardens and Domain Trust, Mrs Macquaries Road, Sydney, NSW 2000, Australia

**Keywords:** Biogeography, bracken, ferns, Galapagos, hybridization, islands, nuclear genes, phylogeny, *Pteridium*

## Abstract

Isolated oceanic islands are characterized by patterns of biological diversity different from that on nearby continental mainlands. Isolation can provide the opportunity for evolutionary divergence, but also set the stage for hybridization between related taxa arriving from different sources. Ferns disperse by haploid spores, which are produced in large numbers and can travel long distances in air currents, enabling these plants to become established on most oceanic islands. Here, we examine the origins and patterns of diversity of the cosmopolitan fern genus *Pteridium* (Dennstaedtiaceae; bracken) on the Galapagos Islands. We use nucleotide sequences from two plastid genes, and two nuclear gene markers, to examine phylogeography of *Pteridium* on the Galapagos Islands. We incorporate data from a previous study to provide a worldwide context. We also sampled new specimens from South and Central America. We used flow cytometry to estimate genome size of some accessions. We found that both plastid and nuclear haplotypes fall into two distinct clades, consistent with a two-diploid-species taxonomy of *P. aquilinum* and *P. esculentum*. As predicted, the allotetraploid *P. caudatum* possesses nuclear haplotypes from both diploid species. Samples from the Galapagos include *P. esculentum* subsp. *arachnoideum*, *P. caudatum* and possible hybrids between them. Multiple *Pteridium* taxa were also observed growing together at some sites. We find evidence for multiple origins of *Pteridium* on the Galapagos Islands and multiple origins of tetraploid *P. caudatum* throughout its range in Central and South America. We also posit that *P. caudatum* may include recent diploid hybrids, backcrosses to *P. esculentum*, as well as allotetraploid plants. The Galapagos Islands are positioned close to the equator where they can receive dispersing propagules from both hemispheres. This may partly explain the high levels of diversity found for this cosmopolitan fern on these islands.

## Abstract (precis)

Isolated oceanic islands are characterized by patterns of biological diversity different from those on continents. Nucleotide sequences from chloroplast and nuclear genes were used to examine the origins and diversity of the cosmopolitan fern genus *Pteridium* on the Galapagos Islands. We found evidence for multiple origins of the widespread allotetraploid *P. caudatum*. We also show that the Galapagos Islands are home to *P. caudatum* as well as diploid *P. esculentum* subsp. *arachnoideum* and possible hybrids between the two. Haplotype diversity indicates that *Pteridium* has colonized the islands multiple times and probably from diverse mainland sources.

## Introduction

Oceanic islands provide an ideal biological setting for evolutionary change and thus for the study of evolutionary processes. On islands that are distantly isolated from continents, there can be an increased opportunity for organisms to diverge genetically from those in the original source populations. Furthermore, remote islands can act as a sink for individuals of the same species (or closely related species) arriving from more than one original source, thereby setting the stage for hybridization, increased genetic diversity, or both. Angiosperms and gymnosperms colonize islands most often via seeds, which contain diploid embryos. This is contrasted in ferns (monilophytes) and lycophytes, which are dispersed by haploid spores. In many cases, spore-bearing plants require two spores, and subsequently two gametophytes, for successful establishment (Soltis and Soltis 1987; Soltis *et al*. 1988). Moreover, spore-dispersed plants appear to have high levels of gene flow relative to seed plants (Soltis and Soltis 1987), presumably because spores are smaller and more easily transported in the air than most seeds. An increased dispersal potential can result in colonization of new areas from multiple spore sources, and may involve an increased opportunity for hybridization relative to seed plants. Here, we explore this possibility as we assess the origins and diversity of the cosmopolitan fern genus *Pteridium* on the Galapagos Islands.

The Galapagos Islands are a group of ∼14 main islands and ∼100 rocks or islets, ∼1000 km west of mainland Ecuador (Snell *et al*. 1996). The islands vary in age ranging from ∼0.7 to ∼4.2 million years since emergence above sea level (White *et al*. 1993). However, additional evidence points to a much older archipelago existing at the same spot prior to the current emergence (Christie *et al*. 1992). Rassmann (1997) cited this idea to explain why estimates of some lineage ages are more than 5 million years old. The relative isolation of the islands from the nearest mainland coasts (Ecuador and Costa Rica) has resulted in a high level of endemism. There are estimated to be 236 endemic plant species on the islands (Tye and Francisco-Ortega 2011). Origins of the Galapagos flora are now thought to be quite diverse and include northern and southern Andes and other parts of South America, as well as Central America and the Caribbean (Tye and Francisco-Ortega 2011). The wide range of elevations and rainfall patterns results in a diversity of ecological habitats (Itow 2003).

*Pteridium* is a worldwide genus that has been treated from as few as one species to >20. There are several taxonomic challenges in the genus, one of which is the high level of variability and phenotypic plasticity for morphological characters, including those that are used for taxonomic treatments. Furthermore, regional and local treatments often do not incorporate the context of variation that is seen at the global scale. A few authors have examined *Pteridium* in a worldwide context. For example, Ching (1940) considered the genus to comprise 6 species, whereas a year later, Tryon (1941) treated *Pteridium* as a single species with 2 subspecies and 12 varieties. Page (1976) reviewed information on geographic variation and concluded that there is probably more than one species, but he made no formal taxonomic changes. More recently, in a series of articles (Thomson 2000, 2012; Thomson *et al*. 2008), Thomson has recognized two main diploid species: *P. aquilinum* (corresponding to Tryon's subspecies *aquilinum*) from Europe, North America, Asia and Africa and *P. esculentum* (corresponding approximately to Tryon's subspecies *caudatum*). *Pteridium esculentum* is treated by some authors as two species: *P. esculentum* in Australia and New Zealand and *P. arachnoideum* in South America (see, for example, Schwartsburd *et al*. 2014), whereas others treat *esculentum* and *arachnoideum* as subspecies of *P. esculentum* (Thomson 2012; Zhou *et al*. 2014), a system we follow here. Regardless of rank assignment, evidence for two main clades of *Pteridium* includes analyses of plastid DNA variation (Der *et al*. 2009; Zhou *et al*. 2014). Further, several hybrids and allotetraploids have been examined (Thomson and Alonso-Amelot 2002; Zhou *et al*. 2014). Der *et al*. (2009) noted that development of nuclear genomic markers would be critical for establishing the origins of hybrid taxa and for other systematic studies of *Pteridium*.

South America is home to two main *Pteridium* taxa: diploid *P. esculentum* subsp. *arachnoideum* and allotetraploid *P. caudatum*, the latter a hybrid between *P. esculentum* from South America and *P. aquilinum* from North America (Thomson and Alonso-Amelot 2002). Tetraploidy was inferred on the basis of Feulgen cytometry (Tan and Thomson 1990) and spore size and guard cell length (Thomson and Alonso-Amelot 2002). The hybrid origin of *P. caudatum* is further supported by the additive pattern of DNA markers from *P. aquilinum* and *P. esculentum* (Thomson 2000; Thomson and Alonso-Amelot 2002). Additional characters that can be used to distinguish *P. caudatum* from *P. esculentum* subsp. *arachnoideum* in South America include the presence of gnarled trichomes between veins abaxially (Thomson and Martin 1996) and free laminar lobes on *P. esculentum*. An additional taxon was recently described from north eastern Brazil (Schwartsburd *et al*. 2014).

Most chromosome counts of *Pteridium* show 2*n* = 104 (Page 1976; Sheffield *et al*. 1989; Thomson 2000; Tindale and Roy 2002; Bainard *et al*. 2011), with other complements, such as triploidy (Sheffield *et al*. 1993), assumed to be rare. One count of 2*n* = 52 from Spain (Löve and Kjellqvist 1972) has not been corroborated despite resampling from the same area (Sheffield *et al*. 1989). Jarrett *et al*. (1968) reported the first observation of cytological variation in the genus, with a count of 2*n* = 208 (tetraploid) for one sporophyte of *Pteridium* from the Galapagos Islands. This report has been the motivation for previous as well as the current focus on *Pteridium* from these islands. Klekowski (1973) used gametophytes grown from spores collected from the islands to examine the ability to self-fertilize and cross with *Pteridium* from other sources. The results demonstrated that bracken from Hawaii (*P. aquilinum*) and samples from the Galapagos Islands were interfertile with *Pteridium* from Central and South America. However, Hawaiian and Galapagos *Pteridium* were intersterile with each other.

Recent examination of *Pteridium* collections from the Galapagos islands suggests that more than one taxon is present on Galapagos. We set out to examine Galapagos *Pteridium* with the following objectives:

1. To examine the origins of *P. caudatum* on both islands and mainland.
2. To determine how many *Pteridium* taxa are on the Galapagos Islands.
3. To examine whether different *Pteridium* taxa occupy different islands, or elevations on the Galapagos Islands.
4. To examine the possible mainland origins of Galapagos *Pteridium*.

## Methods

**Figure 1.**
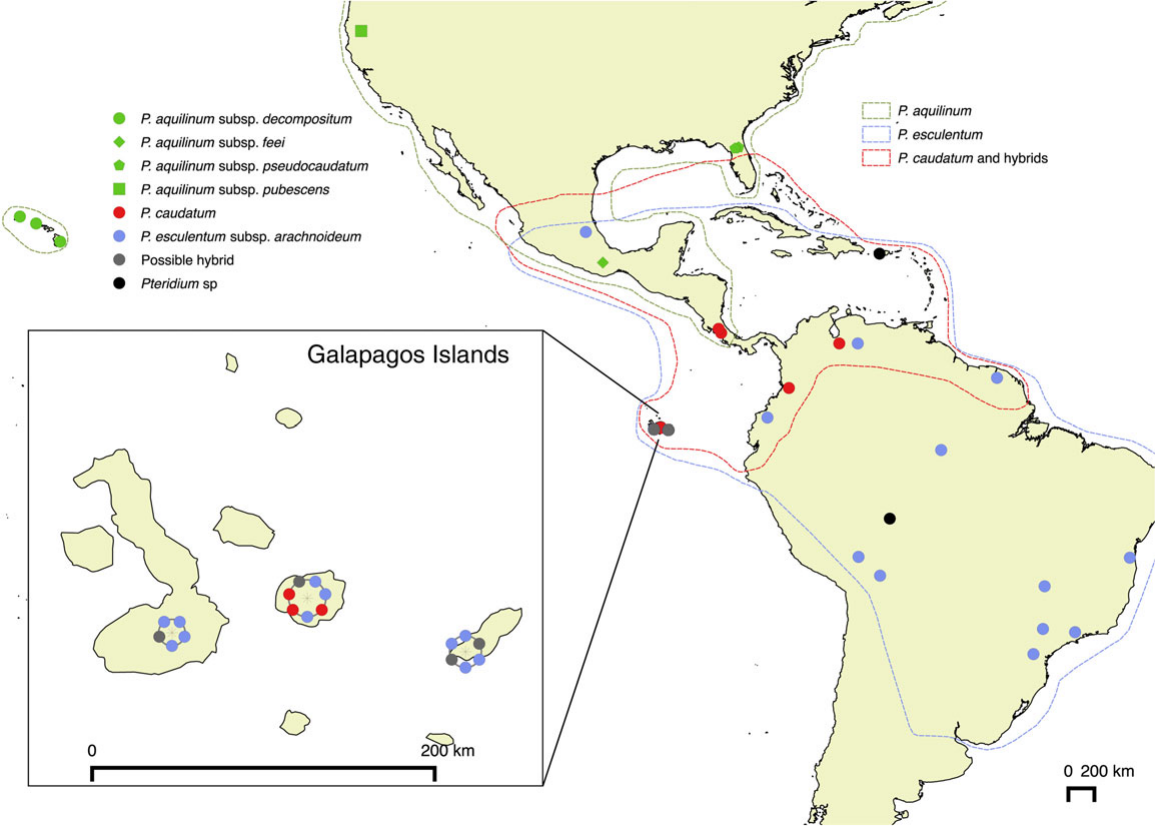
Map of America showing locations of *Pteridium* sampled for this study. Inset shows details of Galapagos Islands. Approximate taxon boundaries are based on Tryon (1941), Tryon and Tryon (1982) and Mickel and Smith (2004).

We sampled 17 *Pteridium* from three of the Galapagos Islands (Fig. 1; Table 1): Santa Cruz, Isabela and San Cristobal. We also scouted on Floriana, but were unable to locate any *Pteridium* on that island. At each site, we collected expanding frond segments onto silica gel, and collected intact fronds for herbarium specimens, deposited at the herbarium of the Charles Darwin Research Station (CDS). In addition, we included a selection of DNA samples from the plastid gene study of Der *et al*. (2009) to obtain nuclear gene sequences. We selected representatives of each of the major plastid clades of Der *et al*. (2009). We also included additional *Pteridium* samples from the mainland of Central and South America, and outgroups *Histiopteris*, *Blotiella* and *Paesia* (Table 1).

**Table 1.** Voucher and locality information for samples used in this study. Code (as used in tree figures) indicates collector and number, with full name in parentheses when abbreviated in code. ‘Possible hybrids’ are likely to be between *P. esculentum* subsp. *arachnoideum* and *P. caudatum*.

Code	Herbarium	Taxon	Country	Island/province/state	Latitude (°)	Longitude (°)	Elevation (m)
Wolf 1001	CDS	*P. esculentum* subsp. *arachnoideum*	Ecuador	Santa Cruz	−0.63	−90.38	592
Wolf 1002	CDS	*P. caudatum*	Ecuador	Santa Cruz	−0.66	−90.40	420
Wolf 1003	CDS	*P. esculentum* subsp. *arachnoideum*	Ecuador	Santa Cruz	−0.64	−90.33	874
Wolf 1004	CDS	*P. esculentum* subsp. *Arachnoideum*	Ecuador	Santa Cruz	−0.65	−90.33	732
Wolf 1005a	CDS	Possible hybrid	Ecuador	Santa Cruz	−0.66	−90.33	580
Wolf 1005c	CDS	*P. caudatum*	Ecuador	Santa Cruz	−0.66	−90.33	580
Wolf 1006	CDS	*P. caudatum*	Ecuador	Santa Cruz	−0.67	−90.32	476
Wolf 1007	CDS	*P. esculentum* subsp. *arachnoideum*	Ecuador	Isabela	−0.81	−91.09	1009
Wolf 1008	CDS	*P. esculentum* subsp. *arachnoideum*	Ecuador	Isabela	−0.83	−91.09	1006
Wolf 1009	CDS	*P. esculentum* subsp. *arachnoideum*	Ecuador	Isabela	−0.84	−91.09	822
Wolf 1010	CDS	*P. esculentum* subsp. *arachnoideum*	Ecuador	Isabela	−0.84	−91.07	627
Wolf 1011	CDS	*Possible hybrid*	Ecuador	Isabela	−0.85	−91.04	405
Wolf 1012	CDS	*P. esculentum* subsp. *arachnoideum*	Ecuador	San Cristobal	−0.91	−89.55	381
Wolf 1013	CDS	*P. esculentum* subsp. *arachnoideum*	Ecuador	San Cristobal	−0.90	−89.48	683
Wolf 1014	CDS	Possible hybrid	Ecuador	San Cristobal	−0.90	−89.48	676
Wolf 1015	CDS	*P. esculentum* subsp. *arachnoideum*	Ecuador	San Cristobal	−0.90	−89.52	739
Wolf 1016	CDS	*P. esculentum* subsp. *arachnoideum*	Ecuador	San Cristobal	−0.90	−89.53	544
Wolf 1017	CDS	Possible hybrid	Ecuador	San Cristobal	−0.90	−89.53	544
Wolf 1018	UTC	*P. aquilinum* subsp. *decompositum*	USA	Hawaii	19.43	−155.28	1247
Wolf 1019	UTC	*P. aquilinum* subsp. *pseudocaudatum*	USA	Florida	29.63	−81.92	42
AL 147 (A. Larsson)	DUKE	*P. aquilinum* subsp. *feei*	Mexico	Oaxaca	17.17	−96.60	2660
IJ 786 (Jiménez)	LPB, UC	*P. esculentum* subsp. *arachnoideum*	Bolivia	Franz Tamayo	−14.62	−68.95	2350
IJ 1245 (Jiménez)	LPB, UC	*P. esculentum* subsp. *arachnoideum*	Bolivia	Ayopaya	−16.65	−66.62	2750
IJ 2048 (Jiménez)	LPB, UC	*Pteridium* sp*.*	Bolivia	Federico Román	−10.48	−65.57	140
Wolf 1020	UTC	*P. caudatum*	Costa Rica	San Jose	9.56	−83.80	2270
Wood 15788	HAW	*P. aquilinum* subsp. *decompositum*	USA	Hawaii	22.15	−159.65	1280
Wolf 1023	HAW	*P. aquilinum* subsp. *decompositum*	USA	Hawaii	21.40	−157.89	419
Worthington 35231	DUKE	*Pteridium* sp*.*	Puerto Rico	Ponce	18.13	−66.68	792
JJdG 14388 (de Granville)	NSW 729390	*P. esculentum* subsp. *arachnoideum*	French Guiana	Saint-Laurent-du-Maroni	4.70	−53.97	480
Matos 231	NY 01198119	*P. esculentum* subsp. *arachnoideum*	Brazil	Bahia	−14.71	−39.60	700
Ortiz 497	NY 00089157	*P. esculentum* subsp. *arachnoideum*	Ecuador	Esmeraldas	0.40	−78.80	1925
Delprete 10293	NY 01019119	*P. esculentum* subsp. *arachnoideum*	Brazil	Goias	−17.80	−48.82	1150
Prado 2351	SP	*P. esculentum* subsp. *arachnoideum*	Brazil	Paranà	−25.14	−50.03	1000
Prado 2337	SP	*P. esculentum* subsp. *arachnoideum*	Brazil	São Paulo	−22.77	−45.53	1888
Wolf 795	UC 1622577	*H. incisa*					
Wolf 387	UTC	*P. scaberula*					
Wolf 376	UTC	*B. pubescens*	La Réunion

### Morphology

Preliminary morphological analysis of samples was undertaken independently of molecular studies. Photographs of fresh unpressed pinnae supplemented by macrophotographs of the abaxial surface of individual ultimate segments were used for the examination of gross features of laminal dissection, presence/absence of free laminal lobes on pinna and pinnule axes, and abaxial laminal indumentum.

We used small subsamples of each accession comprising one or two dried pinnules for more detailed microscopic study following Thomson and Martin (1996). We examined the indument of abaxial pinnulet and segment midveins, determined presence versus absence of gnarled intervein trichomes, measured false-indusial width, estimated the number of cells per millimetre along the outer margin of the false indusium and measured stomatal guard cell length. Taxonomic designation was based on previous descriptions of the characters we used (Thomson 2000, 2012; Thomson and Alonso-Amelot 2002; Thomson *et al*. 2008).

### DNA sequencing

DNA was extracted from fresh, desiccated or herbarium tissue using the DNeasy Plant Mini kit (QIAGEN, Valencia, CA, USA), following the manufacturer's protocol.

The plastid markers *trnS–rpS4* (spacer + gene) and *rpL16* intron were amplified in 25 μL polymerase chain reactions (PCRs) using the fern-specific primers published in Small *et al*. (2005). However, the complete plastid genome sequence of *P. aquilinum* (GenBank accession NC_014348) was used to redesign the *rpl16* reverse and the *trnS* primers (names now with ptaq suffix; see Table 2).

**Table 2.** Primer sequences for PCR and DNA sequencing. Suffix ‘ptaq’ denotes primers designed in this study.

Primer name	Primer sequence, 5′–3′
rpl16_r_ptaq	TCCTCTATGTTGCTTACGATAT
trns_gga_ptaq	CTACCGAGGGTTCAAATCCCTC
SQD_r2_ptaq	CCTTTGCCATAAACTGTAAGGGGGTG
EMSQD1E1F6	GCAAGGGTACHAAGGTHATGATCATAGG
ApPEFP_f25_ptaq	AATGCTCTAAGTCATTGTTACCGATC
ApPEFP_C4218_r7	TTGTAAATCTCTGTRTCRGATGYYGT
rps4_int_f1	CAGATTACTGAAAAACTAGC
rps4_int_r1	AGAAGAGCGAAAGGGTTC
rpl16_int_f1	GCGAAGCTGAAAACGATGCC
rpl16_int_r1	GTTCCATTTCTAAATAGCGG

Nuclear primers were based on those of Rothfels *et al*. (2013). We chose two nuclear genes *SQD1* (Region 1) and *ApPEFP_C* (Region 2), redesigning (now with suffix _ptaq) the forward *ApPEFP_C* and *SQD1* reverse, based on *Pteridium* sequences from a study of the transcriptome (Der *et al*. 2011). *SQD1* encodes sulfoquinovosyldiacylglyerol 1 involved in the biosynthesis of sulfolipids and *ApPEFP_C* encodes an appr-1-p processing enzyme family protein, ADP-ribose-1-monophosphatase (Appr-1-pase), a ubiquitous cellular processing enzyme. The PCR primer sequences (Table 2) used were as follows: SQD_r2_ptaq combined with EMSQD1E1F6, and ApPEFP_f25_ptaq combined with ApPEFP_C4218_r7. Polymerase chain reaction conditions followed Der *et al*. (2009), annealing at 56.5 °C for the plastid and nuclear genes. For sequencing, we used all PCR primers plus new internal primers (Table 2) for the two plastid genes: rps4_int_f1, rps4_int_r1, rpl16_int_f1 and rpl16_int_r1. In many samples, the two nuclear gene amplicons contained multiple haplotypes. To sequence each haplotype separately, we cloned the PCR products using the StrataClone PCR Cloning Kit (Agilent Technologies, Santa Clara, CA, USA). DNA sequences were assembled and edited using Sequencher 4.6 (Gene Codes Corporation, Ann Arbor, MI, USA).

### Phylogenetic analysis

Sequences were aligned with MAFFT version 7.215 using the L-INS-i algorithm for accurate alignments. Newly generated sequences for the plastid genes *rps4* and *rpl16* were combined with data from Der *et al*. (2009; GenBank accession numbers FJ177158–FJ177206 for the *trnS–rps4* spacer + gene and FJ177239–FJ177287 for the rpL16 intron) and concatenated for phylogenetic analysis. Maximum likelihood (ML) phylogenetic inference was performed separately for each nuclear gene and the plastid data with RAxML version 8.1.17 using 100 rapid bootstrap replicates followed by a ML search under the GTRGAMMA model of evolution. Trees were rooted with the three outgroups.

### Flow cytometry

Genome size was determined using flow cytometry. Approximately 0.75 cm^2^ of fresh leaf tissue and 0.5 cm^2^ of standard, *Vicia faba* (26.9 pg; Doležel *et al*. 1998), were co-chopped on a chilled surface using a fresh razor blade in 500 μL of ice-cold extraction buffer (0.1 M citric acid, 0.5 % v/v Triton X-100) (Hanson *et al*. 2005), with 1 % w/v PVP-40 (Yokoya *et al*. 2000). Tissue was chopped into a semi-fine slurry, and the suspension was swirled by hand until the liquid reached a light green tinge. The suspension was poured through a cell strainer (BD Falcon; Becton, Dickinson and Company, Franklin Lakes, NJ, USA). RNaseA (1 mg mL^−1^) and 350 µL of propidium iodide staining solution (0.4 M NaPO_4_, 10 mM sodium citrate, 25 mM sodium sulfate, 50 µg mL^−1^ propidium iodide) were added to 140 µL of filtrate, incubated at 25 °C for 30 min, followed by up to 2 h on ice. The stained solutions were analysed with an Accuri C6 using a 488 nm laser, and 10 000 events were captured per sample. The relative genome size was calculated using the ratio of the mean fluorescent peak of the sample to the internal standard multiplied by the genome size of the standard.

## Results

In general, we found Galapagos *Pteridium* to be highly variable for both morphological and molecular characters. We find evidence of two *Pteridium* taxa plus possible hybrids, and multiple colonization events from different mainland sources.

### Morphology

*Pteridium caudatum* and *P. esculentum* can be distinguished morphologically by a combination of characters (Thomson 2000; Thomson and Alonso-Amelot 2002; Table 3). We inferred that 11 of our samples were clearly *P. e.* subsp*. arachnoideum*, 2 were clearly *P. caudatum* and 4 were difficult to determine and inferred to be possible hybrids. The two *P. caudatum* samples were found at the two lowest sites on Santa Cruz. The possible hybrids were found at the lowest sites on Isabela and San Cristobal and mid-elevation sites on Santa Cruz and San Cristobal. We found different *Pteridium* taxa growing within a kilometre of each other on Santa Cruz and San Cristobal. Samples from one site included one frond that was *P. e. arachnoideum* (Wolf 1005a) and another frond (collected within 1 m of the other) was *P. caudatum* (Wolf 1005c) or a hybrid.

**Table 3.** Typical morphologies for *P. caudatum*, *P. esculentum* subsp. *arachnoideum*, and possible hybrids (or introgressants) between them. Information based on Tryon (1941) and Thomson and Alonso-Amelot (2002).

Determination	Wolf ID #	Free lobes on segment axes	False indusium: width (mm)	False indusium: cells/mm length along margin	Stomatal guard cell length (µm)	Abaxial surface between veins: gnarled trichomes	Abaxial surface: vein indumentum
*P. caudatum*	1002, 1006	Absent	0.3–0.5	∼31	>40	Absent	Glabrous
*P. esculentum* subsp*. arachnoideum*	1001, 1003, 1004, 1007, 1008, 1009, 1010, 1012, 1013, 1015, 1016	Present	0.1–0.3	∼48	<40	Present	Dense fine acicular white hairs, some twisted; fine white arachnoid hairs
Indeterminate: possibly introgressant	1005a	Absent	0.2	64	32.3	Present	Vein hairs less dense than for typical subsp. *arachnoideum*
	1011	Absent	0.4	56	39.1	Present	As for 1005a
	1014	Absent	0.15	48	39.3	Present	As for 1005a
	1017	Absent	0.15–0.2	40	34.3	Absent	As for 1005a

Most of our Galapagos samples of *Pteridium* fell into one of the two distinct categories for stomatal guard cell length: those with a mean below 40 µm and those above 40 µm. Wolf 1002 and Wolf 1006 fall within the range expected for tetraploid *P. caudatum* (Thomson and Alonso-Amelot 2002) and close to the guard cell length (46.5 µm, Thomson 2000) for the Galapagos plant showing 4*n* = 208 (K Sheet H2146/97/1, Jarrett *et al*. 1968), corroborating our morphology-based determination of these samples (Table 3). Ploidy level of the other Galapagos samples studied cannot be determined due to the extended, apparently continuous, series of guard cell lengths represented (Table 3). The wide range of lengths observed suggests that both diploid and triploid levels might be represented. On the basis of morphology, 11 of our samples are *P. esculentum* subsp. *arachnoideum* and 4 may be hybrids carrying genomic elements from outside *arachnoideum*, and may be triploid (Table 3).

### DNA

**Figure 2.**
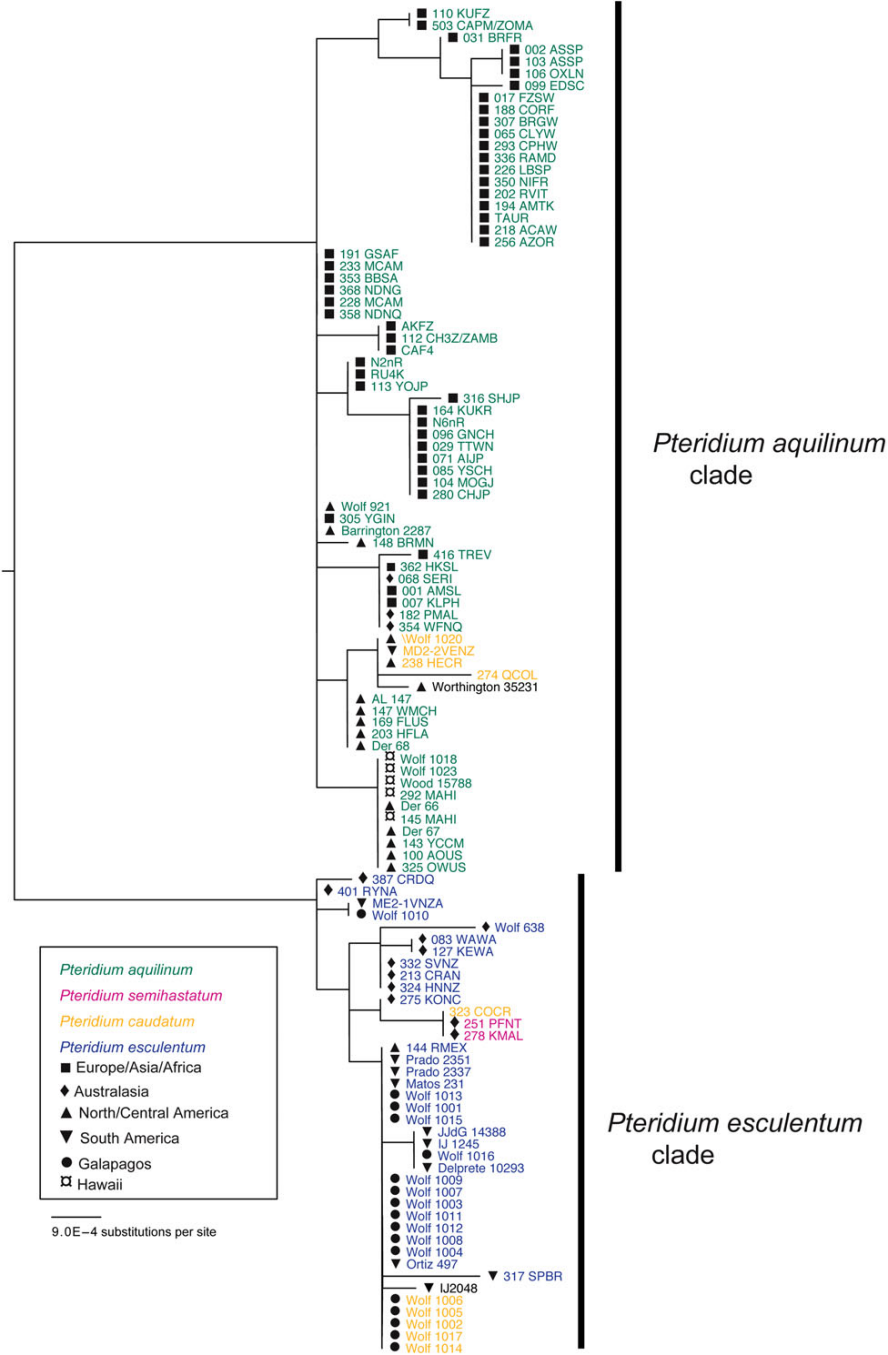
Phylogenetic tree based on the combined plastid gene data set. The tree was rooted with *Blotiella pubescens*, *Paesia scaberula* and *Histiopteris incisa*.

Overall, nucleotide data from the four genes contained 3365 characters of which 100 were phylogenetically informative, and 55 distinguished the *P. aquilinum* clade from the *P. esculentum* clade (Table 4). Phylogenetic analyses of the two plastid genes were congruent, as found previously (Der *et al*. 2009). Thus, alignments of the two plastid genes were concatenated for a combined analysis (Fig. 2). In the three analyses (two plastid genes concatenated, *SQD1* and *ApPEFP_C*), the aquilinum and esculentum clades of *Pteridium* were sister to each other. Table 4 provides the ranges of GenBank accession numbers for each gene and **Supporting Information—File S1** lists the GenBank accession number for each sequence. All trees and associated nucleotide alignments are deposited in TreeBASE (http://purl.org/phylo/treebase/phylows/study/TB2:S18018). We include outgroups for phylogenetic analysis of the nuclear genes. However, inclusion of outgroups in the plastid trees resulted in very short ingroup branch lengths, and is therefore not shown. The tree topology without outgroups is the same as that with outgroups.

**Table 4.** Gene statistics and GenBank accession information.

Gene	Number of characters	Variable characters	Parsimony-informative characters	Differences between aquilinum and esculentum haplotypes	GenBank accession numbers
*ApPEFP_C*	785	51	28	17	KT345729–KT345821
*SQD1*	752	36	26	15	KT345856–KT345898
*rps4*	1036	33	25	11 + 1 indel	KT345822–KT345855
*rpl16*	792	27	21	9 + 2 indels	KT345899–KT345934
Total	3365	147	100	55

We detected a total of 19 aquilinum and 31 esculentum plastid haplotypes. In samples from the Galapagos Islands, we detected 12 plastid haplotypes, one of which has been sampled previously in *P. e. arachnoideum* from Venezuela and Mexico (Der *et al*. 2009). The remaining Galapagos plastid haplotypes were nested within a clade that included haplotypes from Mexico and South America (Fig. 2). All samples of *P. aquilinum* had the expected plastid haplotype, and all new samples from South American *Pteridium*, including those from the Galapagos Islands, had esculentum haplotypes. A specimen from Costa Rica (Wolf 1020), which appears to be *P. caudatum*, had an aquilinum plastid haplotype, as did the single Mexican sample of *P. a. feei* (A. Larsson 147), a taxon not previously included in molecular studies. Previous studies (Thomson *et al*. 2008; Der *et al*. 2009) have noted two 5-bp polymorphic repeats in the *trnS–rps4* spacer. Together, these polymorphisms account for three haplotypes: haplotype C in outgroups and the *P. esculentum* clade, haplotype A in *P. aquilinum* and haplotype B in only European and African *P. aquilinum*.

**Figure 3.**
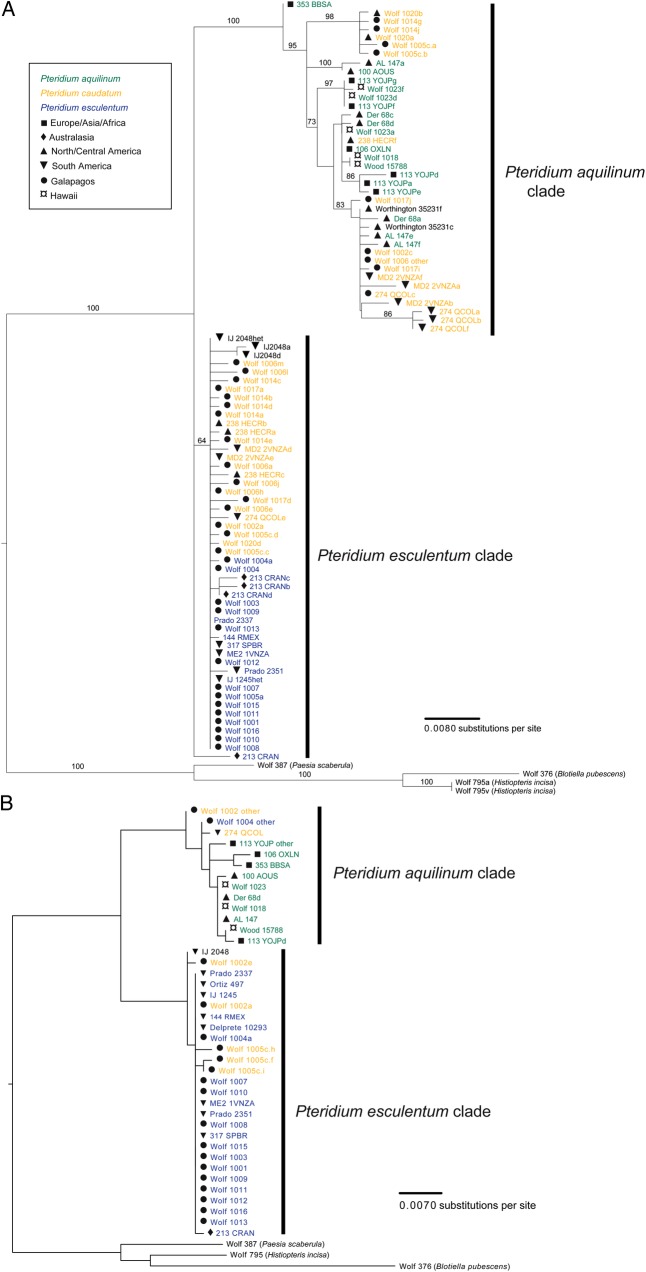
Phylogenetic tree based on the nuclear genes *ApPEFP_C* (A) and *SQD1* (B). Trees were rooted with *B. pubescens*, *P. scaberula* and *H. incisa*.

The two nuclear genes showed a similar pattern of differentiation as the plastid genes: a set of distinct nucleotide differences (15 for *SQD1* and 17 for *ApPEFP_C*) distinguished aquilinum from esculentum haplotypes. For *SQD1*, samples of *P. caudatum* were heterozygous for the above nucleotide positions indicating that they were additive for *P. aquilinum* and *P. esculentum* haplotypes. However, although we were able to sequence a few distinct haplotypes from heterozygous plants, this was largely unsuccessful. Bacterial cells carrying the *SQD1* PCR product appeared to be clumping so that single colonies were usually not single clones and therefore remained heterozygous, despite re-streaking of colonies. We suspect that clumping was a function of partial expression of the gene. Conversely, we were able to clone several haplotypes of *ApPEFP_C* from heterozygous individuals and we found that *P. caudatum* plants indeed possessed both aquilinum and esculentum haplotypes. We detected a total of 12 aquilinum and 13 esculentum haplotypes for *SQD1* (Fig. 3), and 32 aquilinum and 30 esculentum haplotypes for *ApPEFP_C* (Fig. 3). In the samples from the Galapagos Islands, we detected 7 aquilinum and 14 esculentum *ApPEFP_C* haplotypes, and 1 esculentum and 9 aquilinum haplotypes for *SQD1*. Of the six samples that were heterozygous for *SQD1*, we were able to sequence five haplotypes from three individuals. Of the 17 samples that were heterozygous for *ApPEFP_C*, 8 had both aquilinum and esculentum haplotypes, 5 heterozygous samples had only aquilinum haplotypes and 4 samples had only esculentum haplotypes. All Galapagos specimens have esculentum nuclear haplotypes, 11 with an esculentum haplotype only, 10 of which were *P. e. arachnoideum*. Five Galapagos samples have aquilinum and esculentum haplotypes, two of which were *P. caudatum*, two appeared to be hybrids and one was *P. e. arachnoideum*. One specimen (Wolf 1004) appeared to have aquilinum and esculentum haplotypes for *SQD1*, but only aquilinum haplotypes for *ApPEFP_C*. All mainland samples of *P. caudatum* have both aquilinum and esculentum haplotypes. Diploid individuals should have no more than two haplotypes at a locus, whereas tetraploids are expected to have no more than four haplotypes. However, 8 of the 13 heterozygous individuals had extra haplotypes: 3 *P. caudatum* samples with 5, 5 and 7 haplotypes, 1 *P. esculentum* with 6 haplotypes and 4 *P. aquilinum* samples with 3, 3, 3 and 5 haplotypes. In most cases, extra haplotypes differed from others from the same individual by one nucleotide, and at the most three nucleotides. All extra haplotypes appear to be the result of single nucleotide autapomorphies, and we cannot account for extra haplotypes by recombination, whether in plant cells, PCR tubes or during cloning.

### Flow cytometry

We were able to estimate *c*-values for fresh *Pteridium* fronds on a consistent basis, but we were unable to do so for any dry samples, including those in silica gel and herbarium specimens. We estimate haploid genome size (mean ± coefficient of variation) of 15.88 (±0.67) pg for *P. a. decompositum* from Hawaii (Wolf 1018), 16.13 (±0.67) pg for *P. a. pseudocaudatum* from Florida (Wolf 1019) and 29.2 (±2.1) pg for *P. caudatum* from Costa Rica (Wolf 1020). These are consistent with previous estimates for diploid and tetraploid *Pteridium*, respectively (Tan and Thomson 1990; Bainard *et al*. 2011).

## Discussion

In this article, we examined morphological and molecular variation in *Pteridium* from Galapagos Islands. To make inferences about taxonomic variation and possible origins on the islands, we first provided context with other mainland and worldwide samples, including sequences from a previous study (Der *et al*. 2009). We first discuss variation for morphological and molecular characters, followed by the implications for the origins of Galapagos *Pteridium*.

### Molecular data

A growing body of research has examined variation for nuclear-encoded genes within species of ferns (for example see Grusz *et al*. 2009; Nitta *et al*. 2011; Sigel *et al*. 2014). Many studies use nucleotide information from the plastid genome, which appears to be effectively haploid and non-recombining in most plants. Nuclear genes, however, are subject to different processes as a result of chromosomal behaviour at a range of genomic scales. For a single-copy gene, a diploid individual should carry one or two haplotypes (alleles), and a tetraploid can have up to four. Eight of our samples carried more than the expected number based on our estimate of ploidy. Several explanations can account for these results. Polymerase chain reaction and sequencing error could manifest as extra haplotypes within an individual. It is also possible that *SQD1* and *ApPEFP_C* are not strictly single copy in *Pteridium*. Extra haplotypes can occur through several processes including segmental duplication of the chromosomal region carrying the gene, aneuploidy and polyploidy. All measures of genome size and chromosome number in *Pteridium* point to diploid and tetraploid being the most common arrangement. But samples sizes are small and we would benefit from population-level estimates of genome size, especially in areas with multiple species such as Galapagos Islands and areas of Central America. Regardless of the causes of extra haplotypes, they should be explored further. Meanwhile, because the extra haplotypes possessed only a few autapomorphic differences from others, they do not affect the phylogenetic inferences or estimates of origin numbers in this study.

Our only sample from the Caribbean was from a specimen represented by a young frond, and therefore, difficult to identify morphologically. Most descriptions indicate that *P. e. arachnoideum* is throughout the Caribbean, but this sample has aquilinum haplotypes. Future studies would benefit from increase sampling in the Caribbean.

### Morphological variation

*Pteridium* is notorious for its phenotypic plasticity, including morphological variation among fronds within an individual clone and between pinnae on a frond (Tryon 1941; Sheffield *et al*. 1989; Ashcroft and Sheffield 1999; Thomson 2000). Wide morphological variability in *P. caudatum* led Tryon (1941) to recognize several ‘phases’. Ortega (1990) found in Venezuela both typical *P. e. arachnoideum* and a second more compact form lacking free lobes between ultimate segments, while Schwartsburd *et al*. (2014) recognized three morphotypes of *P. e. arachnoideum* from Brazil. The significance and genetic basis of these character suites is yet to be established, but their variation has led to many reports of apparent intermediates between *P. caudatum*, *P. e. arachnoideum* and other taxa (Tryon 1941; Mickel and Beitel 1988; Ortega 1990; Mickel and Smith 2004; Schwartsburd *et al*. 2014).

The relationship between stomatal guard cell length and ploidy level was clearly documented for ferns by Barrington *et al*. (1986) and later established for *Pteridium* (Tan and Thomson 1990; Sheffield *et al*. 1993; Thomson 2000; Thomson and Alonso-Amelot 2002). Guard cell length is quite variable within and between *Pteridium* taxa at the subspecies level (Thomson 2000; Thomson and Alonso-Amelot 2002), and its relationship with ploidy, therefore, requires calibration and validation for each particular comparison, which we followed here.

Most of our samples from the Galapagos Islands (Table 3) had distinct morphological signatures of *P. e. arachnoideum* or *P. caudatum*. However, four samples did not fall clearly into either morphological category (Table 3). Therefore, we infer that the latter samples are possible hybrids between *P. caudatum* and *P. e. arachnoideum*, or the result of a ploidy level other than diploid or tetraploid.

### The origins of *P. caudatum* in South America

Thomson (2000) and Thomson and Alonso-Amelot (2002) first outlined *P. caudatum* as one of the fertile allotetraploids between *P. aquilinum* and *P. esculentum*. Furthermore, these authors speculated that *P. caudatum* has had multiple origins in Central and South America. This hypothesis was supported by analysis of plastid DNA (Der *et al*. 2009), which showed that some *P. caudatum* samples had the *P. aquilinum* plastid DNA, whereas others had that of *P. esculentum*. Here, we provide additional evidence for the hybrid origin of *P. caudatum*; all samples had the additive pattern with both *P. aquilinum* and *P. esculentum* nuclear gene haplotypes. As for many allotetraploids, multiple origins can be inferred (Soltis and Soltis 1991; Ranker *et al*. 1994; Meimberg *et al*. 2009). We detected three *P. aquilinum* plastid haplotypes and seven *P. esculentum* plastid haplotypes among *P. caudatum* accessions. Even more haplotypes are seen in the nuclear DNA, but that is expected because two haplotypes can be transferred in a single origin involving a heterozygous plant. We detected 7 *P. aquilinum* and 12 *P. esculentum* nuclear DNA haplotypes across our *P. caudatum* accessions. Inferring the minimum number of origins is difficult because we do not know how much nucleotide change has occurred since the origin. But given the range of variation found in *P. caudatum*, we can infer at least 8 separate origins among the 11 accessions sampled here. From examination of the phylogenetic trees, it seems that the *P. aquilinum* parent could be *P. a. pseudocaudatum* (Florida and Caribbean), *P. a. latiusculum* (eastern North America) or *P. a. feei* (Mexico). All have similar plastid and nuclear haplotypes so that distinguishing the *P. aquilinum* parent further is challenging. The esculentum parent of *P. caudatum* includes only *P. e. arachnoideum*. However, this taxon is highly variable and probably includes several taxa (Schwartsburd *et al*. 2014). Future sampling should aim to include more samples of *P. e. arachnoideum* from western South America as well as Brazil.

We sampled *P. caudatum* more densely on the Galapagos Islands than the mainland, so it is difficult to determine whether *Pteridium* on the Galapagos Islands is more variable than for an equivalent area on the mainland. However, the variation that we detected indicates that *P. caudatum*, *P. e. arachnoideum* and possible backcrosses can be found in close proximity. Thus, it is possible that plants referred to *P. caudatum* include stable fertile allotetraploids, recently formed allotetraploids, homoploid hybrids between *P. aquilinum* and *P. e. arachnoideum*, and even possible hexaploid hybrids between *P. caudatum* and *P. e. arachnoideum*. Given this level of possible hybridization, we suggest that treating New World *Pteridium* as three species—the diploids *P. esculentum* and *P. aquilinum*, and hybrid *P. caudatum* (in all its manifestations)—represents best the biological situation in the genus (Thomson and Alonso-Amelot 2002; Zhou *et al*. 2014).

### *Pteridium* on Galapagos Islands

We found evidence of *P. e. arachnoideum*, *P. caudatum* and their possible hybrids inhabiting three Galapagos Islands (Fig. 1), often with more than one taxon in close proximity. There was a tendency for *P. caudatum* to be found in lower elevation agricultural areas, but it is not clear if this is because of a habitat preference of *P. caudatum*, or if *P. caudatum* has been introduced with agricultural material. Long distance dispersal via spores is the most likely explanation for colonization of *Pteridium*. In fact, there is evidence of *Pteridium* hybrids in Scotland involving a parent from North America, suggesting transatlantic dispersal of *Pteridium* spores (Rumsey *et al*. 1991). *Pteridium* is highly variable within a relatively small area on the Galapagos Islands, a pattern that has not been observed, to our knowledge, to this extent on the mainland. However, this could be because collections have not been made at the scale used here, or collectors tend to favour specimens that key more easily to one taxon or another, rather than hybrids. If the high variability on the Galapagos Islands is not an artefact, then it could be explained by the location of the Islands. Because the closest mainland areas include both South and Central America, if spores are continually being introduced, then they could easily be coming from multiple sources. This is certainly consistent with the high number of haplotypes on the islands. Introduction from multiple sources has also been inferred for other plant species on the Galapagos Islands (Andrus *et al*. 2009) and for ferns on other island systems (Shepherd *et al*. 2009).

Earlier descriptions of the origin of Galapagos flora attributed the majority of the flora to have a Caribbean origin (Duncan and Hargraves 1984) as the result of ancient vicariance events. Conversely, Porter (1979) hypothesized that the Galapagos flora as a whole was mostly of South American origin. More recently, Tye and Francisco-Ortega (2011) compiled phylogenetic data to infer origins and showed that whereas the largest source (45 % of documented colonization events) was South American, other significant sources included Central America and the Caribbean (12 %), and North America (5 %). *Pteridium* adds an interesting twist to the data because all the above regions appear to be involved, although we do not yet have conclusive evidence for exact sources from North America; they could be from anywhere from Mexico to Florida.

It is unfortunate that we were unable to determine *c*-values for any of our Galapagos samples because this could have been used to test the prediction that putative hybrids between *P. caudatum* and *P. e. arachnoideum* are triploid. Future efforts will be made to sample appropriately for flow cytometry.

## Conclusions

The most striking pattern across worldwide *Pteridium* is the morphological and molecular distinction between the *P. aquilinum* and *P. esculentum* clades. About half of the parsimony-informative molecular characters account for this difference between these two diploid species. How might speciation have occurred in the face of gene flow? One possibility is that initial divergence coincided with the separation of the southern landmasses, which was initiated about 180 million years ago (Scotese 2001) and continued until about 30 million years ago (McLoughlin 2001). However, additional factors would be required to maintain such a pattern of separation between species. One factor evident today is that mostly easterly and westerly prevailing winds operate at the equatorial regions (Oort and Yienger 1996). This would explain the similarities within *P. aquilinum* and within *P. esculentum* (Thomson 2012). However, northerly or southerly wind patterns crossing the equator, such as the inter-tropical convergence zone (Oort and Yienger 1996; Wright *et al*. 2001), are relatively rare. Yet this phenomenon could provide the means necessary for gene flow across the equator to enable hybridization between *P. aquilinum* and *P. esculentum*, thus forming the hybrids *P. caudatum* in South America, and *P. semihastatum* in Australia and tropical Asia (Thomson and Alonso-Amelot 2002).

Our new evidence for *P. caudatum* as a hybrid between diverged diploid species adds to previous examples from studies on ferns. Nitta *et al*. (2011) found evidence of hybrids between geographically distinct clades in the filmy fern genus *Crepidomanes*. Sessa *et al*. (2012) found evidence of rampant hybridization in *Dryopteris*. Furthermore, Rothfels *et al*. (2015) reported hybridization between fern taxa diverged for approximately 60 million years ago. This ability to form hybrids has been attributed to a slower ‘speciation clock’ in plants that lack pre-mating isolation mechanisms that involve biotically mediated fertilization (Rothfels *et al*. 2015). Such patterns are consistent with our findings for *Pteridium* in South America, and particularly on the Galapagos Islands.

In order to gain more resolution on origins of *Pteridium* and its hybrids on the Galapagos Islands, we would need additional genetic resolution. This could best be achieved by sampling at a finer geographic scale, and with many plants per site. In addition to using phylogenetic analysis of nucleotide sequences, it would be useful to include microsatellite loci or single nucleotide polymorphisms (Miller *et al*. 2007; Hohenlohe *et al*. 2010). Given our current data, we found evidence for multiple taxa, multiple origins and likely hybridization on an oceanic archipelago. The results from *Pteridium* add to a growing body of work on the origins of ferns on oceanic islands (Geiger *et al*. 2007) as well as the origins of the general flora of the Galapagos Islands (Tye and Francisco-Ortega 2011). More studies are needed to test whether these results for *Pteridium* extend to spore-bearing plants in general.

## Accession Numbers

All nucleotide sequences used in this manuscript study have been deposited in (and released by) GenBank. GenBank accession numbers are provided in Table 4 and in more detail in **Supporting Information—File S1**. All phylogenetic trees and associated nucleotide alignments are deposited in TreeBASE. These data, including the actual nucleotide sequences, can be accessed by reviewers at http://purl.org/phylo/treebase/phylows/study/TB2:S18018?x-access-code=e0885899a1b8bff199f59defe19bb535&format=html.

## Sources of Funding

This research project was supported by the Mary Gunson Memorial Bequest. C.J.V. was supported by the National Science Foundation Graduate research fellowship programme: DGE-1315138.

## Contributions by the Authors

P.G.W. and J.T. conceived the project, planned the sampling and wrote the manuscript. J.T. conducted morphological and anatomical analyses. M.P.S. led the fieldwork part of the project. C.A.R. conducted all lab work and compiled the data. J.P.D. conducted all phylogenetic analyses and archiving of data. All authors contributed to and approved the manuscript.

## Conflict of Interest Statement

None declared.

## Supporting Information

The following additional information is available in the online version of this article –

**File S1.** List of all haplotypes from all four genes, with corresponding GenBank accession numbers.

Additional Information
