# Supplementary material for: Origins and diversity of a cosmopolitan fern genus on an island archipelago
Source: AoB Plants. 2015 Oct 20;7:plv118. doi: 10.1093/aobpla/plv118 (PMC4662730; doi:10.1093/aobpla/plv118)
Supplement: Additional Information [file supp_7_plv118_index.html]

Origins and diversity of a cosmopolitan fern genus on an island archipelago — Origins and diversity of a cosmopolitan fern genus on an island archipelago — Additional Information 

# Origins and diversity of a cosmopolitan fern genus on an island archipelago

## Additional Information

Additional Information

- Additional Information - csv file
